# Menopausal Status and Physical Performance in Middle Aged Women: A Cross-Sectional Community-Based Study in Northeast Brazil

**DOI:** 10.1371/journal.pone.0119480

**Published:** 2015-03-30

**Authors:** Saionara M. A. da Câmara, Maria Victoria Zunzunegui, Catherine Pirkle, Mayle A. Moreira, Álvaro C. C. Maciel

**Affiliations:** 1 Physiotherapy Department, Universidade Federal do Rio Grande do Norte, Natal, Brazil; 2 School of Public Health, Institut de recherché en santé publique, Université de Montréal, Québec, Canada; 3 Axe Santé publique et pratiques optimales en santé, Centre de recherche du CHUQ, Québec, Canada

## Abstract

**Objective:**

To examine associations between menopausal status and physical performance in middle-aged women from the Northeast region of Brazil.

**Methods:**

Cross-sectional study of women between 40 to 65 years old living in Parnamirim. Women were recruited by advertisements in primary care neighborhood centers across the city. Physical performance was assessed by grip strength, gait speed and chair stands. Menopausal status was determined using the Stages of Reproductive Aging Workshop classification and women were classified in: premenopausal, perimenopausal or postmenopausal. Multiple linear regression analyses were performed to model the effect of menopausal status on each physical performance measure, adjusting for covariates (age, family income, education, body mass index, parity and age at first birth).

**Results:**

The premenopausal women were significantly stronger and performed better in chair stands than perimenopausal and postmenopausal women. Gait speed did not vary significantly by menopausal status. In multivariate analyses, menopausal status remained statistically significant only for grip strength. In fully adjusted analyses, premenopausal women had grip strength mean of 2.226 Kgf (95% CI: 0.361 – 4.091) higher than the postmenopausal group.

**Conclusions:**

This study provides further evidence for the associations between menopause and physical performance in middle-aged women, since grip strength is weaker in peri and postmenopausal women compared to premenopausal, even adjusted for age and other covariates.

## Introduction

Physical performance—lower and upper extremities functioning—may be tested by the ability to perform an action or activity, such as rising from a chair or walking. These tests are integrated markers of aging, influenced by many physiological and clinical characteristics, as well as the social environment [1,2]. At older ages, women on average tend to present lower physical performance than men, suggesting that gender or sex-linked dependent factors across life may influence physical performance [3,4]. Changes in hormonal exposure are one of these possible factors, since loss of muscle mass and strength associated with aging starts at an earlier age in women than in men, around the time of menopause [3,4,5].

Some studies have examined the association between menopause and physical performance, providing evidence that perimenopausal and postmenopausal women have worse physical function than premenopausal women [3,6,7]. However, some contradictory results have also been found as a lack of variation in muscle strength was found among women from different menopausal status [8], thereby making it unclear whether the associations observed between menopause and performance are independent of the changes in performance associated with general aging.

Most studies on this subject were carried out in high-income countries and there is a lack of literature about this association in low-income countries where women differ substantially in terms of life course and reproductive histories. Different distributions for the age at menopause and physical function have been observed when comparing Latin American populations with those in Europe and North America, as well as across Latin American countries. Median age at menopause occurs several years earlier in Latin American women compared to women from Europe and North America [9] with considerable variation across Latin American countries, suggesting that age at menopause is influenced by life course socioeconomic adversity and reproductive history. Social and economic adversity during the life course [10] and reproductive history characterized by early maternal age at first birth and high parity [11] may have strong effects on physical function, but as far as we know, no study has examined the association between menopausal status and physical function taking into account socioeconomic characteristics in a middle or low income setting.

Thus, the aim of this study is to examine the relationship between menopausal status and physical performance in a population of middle-aged women from the Northeast region of Brazil.

## Material and Methods

This study took place in Parnamirim, a city in the Northeast of Brazil, which is located in Natal’s metropolitan region, the capital of Rio Grande do Norte state. This city has around 200,000 inhabitants, distributed across 123.5 km^2^, and is 100% urbanized.

In this paper, we present the baseline data from an ongoing longitudinal research program examining physical performance in middle-aged women. The longitudinal study aims to analyze the influence of hormone levels on sarcopenia (muscle loss) and physical functioning. The present data were collected between April and November of 2013.

### Population and sample

The study population was composed of 40 to 65 year-old women living in Parnamirim.

A convenience sample of five hundred women comprised the baseline study sample. Women were recruited by advertisements in all primary care neighborhood centers across the city. Primary health care is universally funded by the Brazilian government through the Family Health Care program. Exclusion criteria consisted of the following: neurological impairments and painful conditions, such as muscle and joint pain, that might have compromised the physical performance measurement; being a smoker; or having had a double oophorectomy. In addition to the above criteria, for the present study, we excluded women with hysterectomy (n = 93), women that were not able to accurately describe their menstruation pattern (n = 15) and women that did not complete the evaluation (n = 3). Our final sample consisted of 389 women. This sample can be considered representative of the population of Parnamirim’s middle-aged women that undergo natural menopause since they presented similar distribution of education and marital status compared with those in the wider population, according to the last census data.

### Ethics

All participants were informed of the objectives and procedures of the research study at first contact and signed a consent form. The study protocol received ethics approval by the Ethics and Research Committee of the Federal University of Rio Grande do Norte (approval number 387.737).

### Procedures

All women were assessed in a community center in Parnamirim by trained interviewers, using standardized protocols as described below.

### Physical performance

Physical performance was assessed by conducting three tests: grip strength, gait speed and chair stands.

Grip strength: the dominant hand was evaluated with a Jamar dynamometer in the second handle position [12]. The participant was positioned, as recommended by the American Society of Hand Therapists [13], seated with the shoulder fully adducted and neutrally rotated, elbow flexed at 90° and the forearm in a neutral position. The participant was requested to squeeze the dynamometer with maximal isometric effort without any other body movement, for five seconds. The test was performed three times, with a one-minute interval between measures. The mean of these three trials was used for analyses.

For gait speed, a 4-meter walk at the subject’s usual pace was timed. The test was repeated twice with the faster of the two walks used. Gait speed was calculated in meters per second. For the ability to rise from a chair, participants were asked to stand up and sit down five times as quickly as possible with arms folded across their chests and were timed in seconds from the initial sitting position to the fifth standing position. Further details on the administration of these tests have been published in the original papers [14,15].

### Menopausal Status

Menopausal status was determined using the Stages of Reproductive Aging Workshop classification – STRAW [16]. Women were classified in three groups according to self-reported menstruation pattern: premenopausal (regular menses), perimenopausal (irregular menses, with differences on cycle length over seven days or amenorrhea until one year) or postmenopausal (absence of menses for over one year).

### Covariates

Based on previous research, the following potential confounders of the associations under investigation were considered.

Socioeconomic position: Family income and education were considered as possible confounders since they are associated both with physical performance [17] and age at menopause [9]. Family income was categorized, using as a reference the Brazilian minimum monthly wage (MW), which is defined as the lowest remuneration that employers may legally pay workers. Theoretically, this minimum wage should be able to supply the normal needs of food, housing, clothing, hygiene and transport for a family. At the time of the interview, the MW was fixed on an amount of R$ 678.00. However, according to the Statistics and Socioeconomic Studies Department of Brazil (DIEESE) [18], the sufficient minimum salary to cover the basic needs at the time of this research should be four times the value established by the Brazilian government. In our study, family income was thus dichotomized as less than 3 MW and 3 MW or more. The choice of 3 MW was based on what is considered to be a poverty threshold in the Northeastern area of Brazil. Education was assessed as years of schooling and then categorized as: less than basic education (up to seven years), between basic and secondary (more than seven and less than eleven years), and secondary or more (eleven years and over).

Body Mass Index (BMI): BMI is associated to better muscle strength and worse in activities such as gait speed and chair stand [19,20]. BMI (kg/m^2^) was calculated from measured height (m) and weight (kg) and later categorized according to the international classification from the World Health Organization (WHO) as: 18.5 to 24.99 (normal weight), 25.00 to 29.99 (overweight), 30.00 to 34.99 (obese I), ≥35.00 (obese II and III) [21].

Walking: Women were instructed to report how many days and for how long per day they walked for more than 10 minutes without stopping during the last week. The walking variable was dichotomized into less than 90 min/week and 90 min/week or more.

Sitting time: Women were requested to report how much time in a regular day they stay seated to register sedentary activity. This variable was dichotomized in 4 h/day or less and more than 4 h/day.

Reproductive history: Reproductive history has been associated with physical performance [11] and earlier age at menopause [9]. Since more than one fourth of women reported their menarche was at age 13, women were classified into three groups: menarche before age 13, at 13 and after 13 years old. Parity was dichotomized in less than 3 births and 3 births or more and age at first child was categorized into before age 18 and at age 18 years or more; nulliparous women composed a separate category in this variable.

Hypertension: This was assessed by calculating the mean of three blood pressure measurements using a digital sphygmomanometer with the appropriate cuff size. A woman was classified as hypertensive if the mean systolic pressure was 140 mmHg or higher and/or the mean diastolic pressure was 90 mmHg or higher [22].

### Data Analysis

Analyses were carried out using SPSS software, version 20.0 (SPSS, Chicago, IL, USA). First, descriptive statistics for all variables were presented according to menopausal status and analyzed with analysis of variance (ANOVA) and post hoc Tukey test for continuous variables, and with Chi-square tests for comparison of proportions. Means and standard deviations of grip strength, gait speed and chair stands were presented for each category of the independent variables and compared using t-tests. ANOVA models were used to test for linear trends of grip strength and gait speed and time for chair stands according to menopausal categories. The F-statistic to test for linear trend was used to generate p-values. Then multiple linear regression analyses were performed to model the effect of menopausal status on each physical performance measure, adjusted for the covariates that had associations with physical performance with p<0.20 in bivariate analysis (age, family income, education, body mass index, parity and age at first birth). Of note, parity and age at first birth are highly collinear; consequently in the multivariate analyses, we only included nulliparity and age at first birth.

## Results

The characteristics of the sample according to menopausal status are presented in Table 1. Not surprisingly, age was significantly different among the different menopausal groups: premenopausal women were younger, followed by perimenopausal and postmenopausal women and the groups also differed by education and parity. No significant differences by menopausal status were found for income, hypertension, BMI, walking time per week, age at menarche and age at first birth.

**Table 1:** Sample characteristics according to menopausal status (N = 389).

Variables	Premenopausal	Perimenopausal	Postmenopausal	p value
	n (%) or mean (SD)			
**Age (years)**	44.63 (3.36)	48.43 (3.38)	54.47 (5.24)	<0.001^a^
**Family income**				0.501^b^
< 3 MW	71 (68.3)	111 (73.0)	89 (66.9)	
≥ 3 MW	33 (31.7)	41 (27.0)	44 (33.1)	
**Education**				0.001^b^
Less than basic education	34 (32.7)	57 (37.5)	67 (50.4)	
Between basic and secundary	59 (56.7)	60 (39.5)	48 (38.1)	
Secundary or more	11 (10.6)	35 (23.0)	18 (13.5)	
**Hypertension**				0.491^b^
No	89 (85.6)	126 (82.9)	106 (79.7)	
Yes	15 (22.1)	26 (17.1)	27 (20.3)	
**BMI (kg/m** ^**2**^)				0.273^b^
18.5–24.9 (normal)	28 (26.9)	28 (18.4)	27 (20.3)	
25.0–29.9 (overweight)	42 (40.4)	59 (38.8)	59 (44.4)	
30.0–34.9 (obese I)	25 (24.0)	46 (30.3)	27 (20.3)	
≥35.0 (obese II and III)	9 (8.7)	19 (12.5)	20 (15.0)	
**Sitting time per day**				0.642^b^
4h or less	65 (62.5)	94 (61.8)	89 (66.9)	
More than 4h	39 (37.5)	58 (38.2)	44 (33.1)	
**Walking (min/week)**				0.305^b^
<90	61 (58.7)	75 (49.3)	74 (55.6)	
≥90	43 (41.3)	77 (50.7)	59 (44.4)	
**Age at menarche**				0.504^b^
< 13 years old	41 (39.4)	53 (34.9)	40 (30.1)	
13 years old	30 (28.8)	40 (26.3)	37 (27.8)	
> 13 years old	33 (31.7)	59 (38.8)	56 (42.1)	
**Age at first birth**				0.329^b^
No child	6 (5.8)	7 (4.6)	4 (3.0)	
Before 18 years old	26 (25.0)	24 (15.8)	27 (20.3)	
18 years old or more	72 (69.2)	121 (79.6)	102 (76.7)	
**Parity**				0.020^b^
< 3 children	56 (53.8)	78 (51.3)	50 (37.3)	
≥ 3 children	48 (46.2)	74 (48.7)	83 (62.4)	
**Total**	104 (26.7)	152 (39.1)	133 (34.2)	

MW – minimum wages; BMI – Body Mass Index.

a—p value for ANOVA: premenopausal < perimenopausal < postmenopausal

b – p value for Chi-square test.

Table 2 shows the unadjusted analyses for physical performance according to the considered covariates. A clear gradient is observed in grip strength among pre, peri and postmenopausal. On average, the premenopausal women were significantly stronger than perimenopausal women and also stronger than the postmenopausal group. Gait speed did not vary significantly by menopausal status. Lastly, premenopausal women performed better in chair stands when compared with perimenopausal and postmenopausal women.

**Table 2:** Mean levels of physical performance according to covariates.

Factors	Handgrip strength (Kgf)	Gait speed (m/s)	Chair stand (s)
**Total sample**	25.74 (5.54)	0.975 (0.169)	10.12 (2.05)
**Menopausal status**			
Premenopausal	27.50 (5.97)	0.982 (0.157)	9.64 (1.77)
Perimenopausal	25.44 (5.39)	0.971 (0.168)	10.33 (1.82)
Postmenopausal	24.66 (5.04)	0.972 (0.181)	10.24 (2.41)
*p value*	<0.001^a^	0. 865	0.026^a^
**Family income**			
< 3 MW	25.28 (5.51)	0.965 (0.174)	10.34 (2.03)
≥ 3 MW	26.75 (5.49)	0.996 (0.156)	9.63 (2.02)
*p value*	0.016	0.102	0.002
**Education**			
Less than basic education	25.01 (5.25)	0.948 (0.161)	10.44 (2.17)
Between basic and secundary	26.46 (5.78)	0.977 (0.169)	10.03 (1.94)
Secundary or more	25.61 (5.49)	1.033 (0.178)	9.55 (1.87)
*p value*	0.064	0.003^b^	0.011^b^
**Hypertension**			
No	25.80 (5.56)	0.978 (0.172)	10.16 (2.03)
Yes	25.40 (5.49)	0.961 (0.156)	9.94 (2.14)
*p value*	0.596	0.472	0.406
**BMI (kg/m** ^**2**^)			
18.5–24.9 (normal)	24.23 (4.63)	0.982 (0.177)	9.91 (1.66)
25.0–29.9 (overweight)	25.88 (5.82)	0.993 (0.164)	9.99 (2.20)
30.0–34.9 (obese I)	25.75 (5.94)	0.953 (0.173)	10.50 (2.18)
≥35.0 (obese II and III)	27.67 (4.55)	0.944 (0.159)	10.11 (1.73)
*p value*	0.007^c^	0.152	0.193
**Sitting time per day**			
4h or less	25.46 (5.25)	0.966 (0.174)	10.13 (2.14)
More than 4h	26.20 (6.01)	0.990 (0.161)	10.11 (1.89)
*p value*	0.208	0.181	0.927
**Walking (min/week)**			
<90	25.67 (5.38)	0.984 (0.172)	10.40 (2.00)
≥90	25.79 (5.74)	0.964 (0.166)	9.80 (2.05)
*p value*	0.834	0.244	0.004
**Age at menarche**			
Before 13 years old	25.18 (5.43)	0.967 (0.162)	10.29 (1.98)
13 years old	25.79 (5.35)	0.964 (0.172)	10.17 (2.18)
After 13 years old	26.16 (5.77)	0.990 (0.174)	9.92 (2.00)
*p value*	0.334	0.412	0.313
**Age at first birth**			
No child	21.10 (4.88)	0.978 (1.689)	10.18 (2.34)
Before 18 years old	25.44 (4.52)	0.975 (0.165)	10.59 (2.05)
18 years old or more	26.07 (5.71)	0.974 (0.171)	9.99 (2.02)
*p value*	0.001^d^	0.996	0.076
**Parity**			
< 3 children	26.09 (5.26)	0.983 (0.170)	9.84 (1.93)
≥ 3 children	25.35 (5.75)	0.966 (0.169)	10.38 (2.12)
*p value*	0.189	0.336	0.010

MW – minimum wages; BMI – Body Mass Index. Note: greater values are better for grip strength and gait speed, but worse for chair stand.

a: premenopausal ≠ perimenopausal; premenopausal ≠ postmenopausal

b: less than basic education ≠ secondary or more

c: normal weight ≠ obese II and III

d: No child ≠ before 18 years old; No child ≠ 18 years old or more

Strong relationships were found between socioeconomic position and physical performance, with better results for those with higher education and family income. Women with moderate and severe obesity (BMI≥35.0) had higher grip strength compared to those with normal weight (BMI 18.5 to 24.9), without significant differences among the intermediary categories of overweight and light obesity and normal weight. Weekly walking time was associated with shorter time in chair stands but not with gait speed or grip strength. Nulliparous women and women who had their first birth before 18 years of age presented poorer performance for all tests, but the association was significant for grip strength only. Parity was significantly associated with the chair stand test, with better performance for women with less than 3 births. Age at menarche was not associated with any of the three physical performance tests.

Table 3 shows the multiple linear regression results for each performance test. Menopausal status remained statistically significant only for grip strength after adjustment for age, socioeconomic position, obesity and reproductive health covariates. Premenopausal women had significantly greater grip strength than perimenopausal and postmenopausal women; perimenopausal women had greater grip strength than postmenopausal women, but this difference was not significant.

**Table 3:** Adjusted multiple regression models for performance tests: grip strength, gait speed and chair stand.

		Grip Strength (Kgf)				Gait speed (m/s)				Chair Stand (s)			
	Variable	β	CI 95%		P value	β	CI 95%		P value	β	CI 95%		P value
			Lower	Upper			Lower	Upper			Lower	Upper	
**Model 1**	**Menopausal status**												
	Premenopausal	2.845	1.442	4.247	0.000	0.010	-0.034	0.054	0.659	-0.602	-1.139	-0.066	0.028
	Perimenopausal	0.778	-0.497	2.052	0.231	-0.001	-0.041	0.039	0.956	0.082	-0.397	0.562	0.736
	Postmenopausal	0				0				0			
**Model 2**													
	Premenopausal	2.058	0.137	3.978	0.036	-0.004	-0.064	0.057	0.909	-0.388	-1.114	0.338	0.294
	Perimenopausal	0.294	-1.214	1.802	0.702	-0.009	-0.056	0.038	0.697	0.214	-0.352	0.779	0.458
	Postmenopausal	0				0				0			
**Model 3**													
	Premenopausal	2.012	0.101	3.924	0.039	-0.001	-0.061	0.058	0.971	-0.432	-1.148	0.284	0.236
	Perimenopausal	0.408	-1.099	1.915	0.595	-0.014	-0.061	0.033	0.555	0.225	-0.334	0.784	0.428
	Postmenopausal	0				0				0			
**Model 4**													
	Premenopausal	2.226	0.361	4.091	0.019	-0.008	-0.067	0.052	0.768	-0.489	-1.202	0.223	0.178
	Perimenopausal	0.292	-1.180	1.765	0.697	-0.013	-0.060	0.034	0.595	0.254	-0.304	0.812	0.371
	Postmenopausal	0				0				0			

CI: Confidence Interval.

Model 1: Unadjusted. Model 2: Adjusted for age only. Model 3: Adjusted for age, family income and education. Model 4: Adjusted for age, family income, education, body mass index, parity and age at first birth.

## Discussion

According to the results, there is evidence to suggest inferior physical performance associated with menopause, since peri and postmenopausal women presented worse results in grip strength and chair stand performance compared to premenopausal women. The association between grip strength and menopausal status was maintained after extensive adjustments, including for age, socioeconomic and reproductive factors. However, we did not find significant differences in physical performance when comparing perimenopausal and postmenopausal women. No significant differences in gait speed according to menopausal status were found.

Compared to the results of previous investigations of physical function using objective assessments among women according to menopausal status, our findings present some divergences. Bassey et al. [8] assessed handgrip, quadriceps, and leg extensor muscle strength in English middle-aged women and concluded that menopausal status was not a significant contributor to the explained variance of any muscle measurement, even after consideration of other factors that were significant. However, this study did not use standard definitions of menopausal stages and presented small groups for some categories (N = 15; N = 33), which could have reduced the power of the analysis. A study by Cooper et al. [3] found that postmenopausal women did have weaker grip strength than premenopausal women, but this difference was not statistically significant. These authors studied a British population (the British birth cohort study) without any confounding effect by age since all participants were born in the same week of the same year and had their physical performance assessed at the same age. Although our study had adjusted the results for age, some residual confounding factor is possible and this could be one reason for the difference between both studies. Also, the classification of the perimenopausal category should be considered to explain the differences in the results found. The British study considered women in perimenopausal status if their menses had ceased for at least 3 months. According to the STRAW staging system, widely considered the gold standard for characterizing reproductive aging through menopause [16], an increased variability in menstrual cycle length for 7 days until 60 days is sufficient to be characterized as early perimenopausal status. The late perimenopausal status is marked by the occurrence of amenorrhea of 60 days or longer. Based on the STRAW staging system, it is possible that the premenopausal group in Cooper’s study also contained perimenopausal women, leading to some misclassification.

However, similar to our results, Cheng et al. [7] studied a Taiwanese population and found that muscle strength and balance ability were poorer in postmenopausal women. Their sample was composed of women living in a location where most people were engaged in farming activities or small businesses and had low education. Also, the results of the SWAN longitudinal study [23] indicated that transition through menopause is associated with a decline in pinch strength. That study was based on a population composed of African-American and Caucasian women living in an area from Chicago (IL), where the ranges of socioeconomic position are broad and overlapping for both racial groups.

Interestingly, although grip strength and chair stands were significantly associated with menopausal status, only grip strength remained significant after adjustment. These results suggest that the relation between menopause and physical performance occurs through the musculoskeletal system rather than other body systems, as discussed before by Cooper et al. [3]. The chair stand test is considered an indirect measurement of lower limbs strength, but as is the case for the gait speed test, the integrity of the cardiovascular, respiratory and vestibular systems is also important to appropriately perform these two tasks and menopause may not exert a strong influence on these systems.

Gait speed was not related to menopausal status in our study sample. It is possible that gait speed variance in the examined age group is not large enough to detect small changes with a relatively small sample. In older populations, inability or slowness to rise from a chair appears earlier than slowness in regular walking. Thus, it is not surprising to observe that changes in chair stand times are more variable than changes in gait speed around menopause.

The relation between grip strength and menopause could be explained by the hypothesis that menopause transition and the subsequent decline in estrogen may play a role in muscle mass and strength loss. Previous studies have already reported positive relationships between estrogen levels and muscle mass [24,25] and muscle strength [26,27]. Estrogen may have an anabolic effect on muscle by the stimulation of IGF-1 receptors and estrogen receptors that are present in human muscles (expressed at the mRNA level); however, these hypotheses are not well established and more research is necessary [5]. Moreover, some important factors not specific to menopause but which can be exacerbated by changes in menopausal status may contribute to decline in muscle mass and strength with menopause; for example, physical inactivity, less protein intake and oxidative stress [5,28]. These factors are more common among people living in lower income settings and may explain why changes during menopause are significant in our sample and the Taiwanese study [7], but non-significant in the British studies [3,8]. This is in agreement with the overall lower means of grip strength observed in this sample compared with the UK study of women around menopause. Previous studies have reported associations between socioeconomic position during life course and health in adulthood with consistent evidence that the socioeconomically disadvantaged have poorer physical function than the more advantaged [10,17,29–31]. Disability in older ages may begin early in midlife, especially for women with low socioeconomic position [32]. Social inequalities are still strong in Brazil, particularly in the Northeast area, and factors such as personal health behavior, health care access, environmental health exposures and psychosocial stressors may have affected these women across life. According to the life course epidemiology framework, low income and low educated women may reach menopause with a poor physiologic reserve and their losses on muscle performance during menopause would be relatively larger compared to high income and well educated women.

### Strengths and limitations

As far as we know this is the first study investigating the association between physical performance and menopause in a Latin-American population of middle-aged women. Our results highlight the importance of considering socioeconomic conditions when studying this relation. The cross-sectional design of the study limits causal inferences and the results could have been influenced by cohort effects, since opportunities for education and contraception have increased in Brazil in recent decades. Women in postmenopausal groups were less educated and had more children than the younger women of the other groups, but the relation between grip strength and menopause remained even after adjustment for parity and education. Additionally, although women in this sample were recruited through convenience sampling, the socioeconomic characteristics are similar to other samples of community-based studies in the area [31,33] and this sample also presents similar socioeconomic characteristics to the population of Parnamirim’s women according to the last census data. Finally, although some information was collected by self-reporting, and particularly menopausal status for which bias can occur for those with lower education, self-reported questionnaires on menstrual bleeding patterns are the usual method in the literature [6,9,34]. Furthermore, objective and validated measures of physical performance were used.

## Conclusions

The present study showed that the transition through menopause is associated to physical performance in a population of Latin-American women. Weaker grip strength was found in peri and postmenopausal women compared to premenopausal women. Menopause seems to be related more strongly with the musculoskeletal system than other body systems, such as the cardiovascular and vestibular systems, as there was no relation found between menopausal status and gait speed and associations between menopausal status and timed chair stand were attenuated after adjustment for socioeconomic position, body weight, physical activity and reproductive history.

## References

[1] Guralnik JM, Winograd CH (1994) Physical performance measures in the assessment of older persons . Aging (Milano) 6(5):303–5. 7893776 10.1007/BF03324256

[2] Verbrugge LM, Jette AM (1994) The disablement process. Soc. Sci. Med. 38(1):1–14. 8146699 10.1016/0277-9536(94)90294-1

[3] Cooper R, Mishra G, Clennell S, Guralnik J, Kuh D (2008) Menopausal status and physical performance in midlife: findings from a British birth cohort study. Menopause 15(6):1079–85. 10.1097/gme.0b013e31816f63a3 18520694 PMC3182533

[4] El Khoudary SR, McClure CK, VoPham T, Karvonen-Gutierrez CA, Sternfeld B, et al (2014) Longitudinal Assessment of the Menopausal Transition, Endogenous Sex Hormones, and Perception of Physical Functioning: The Study of Women’s Health Across the Nation. J Gerontol A Biol Sci Med Sci 69:1011–7. 10.1093/gerona/glt285 24465026 PMC4158400

[5] Maltais ML, Desroches J, Dionne IJ (2009) Changes in muscle mass and strength after menopause. J Musculoskelet Neuronal Interact 9(4):186–197. 19949277

[6] Sowers M, Pope S, Welch G, Sternfeld B, Albrecht G (2001) The association of menopause and physical functioning in women at midlife. J. Am. Geriatr. Soc. 49(11):1485–92. 11890587 10.1046/j.1532-5415.2001.4911241.x

[7] Cheng MH, Wang SJ, Yang FY, Wang PH, Fuh JL (2009) Menopause and physical performance—a community-based cross-sectional study. Menopause 16(5):892–6. 10.1097/gme.0b013e3181a0e091 19455071

[8] Bassey EJ (1996) Lack of variation in muscle strength with menstrual status in healthy women aged 45–54 years: Data from a national survey. Eur. J. Appl. Physiol. Occup. Physiol. 73(3–4):382–386. 8781873 10.1007/BF02425503

[9] Vélez MP, Alvarado BE, Lord C, Zunzunegui M-V (2010) Life course socioeconomic adversity and age at natural menopause in women from Latin America and the Caribbean. Menopause 17(3): 552–559. 20464784

[10] Birnie K, Cooper R, Martin RM, Kuh D, Sayer AA, et al (2011) Childhood socioeconomic position and objectively measured physical capability levels in adulthood: a systematic review and meta-analysis. PLOS ONE 6(1): e15564 10.1371/journal.pone.0015564 21297868 PMC3027621

[11] Pirkle C, Sousa ACPA, Alvarado B, Zunzunegui MV (2014) Early maternal age at first birth is associated with chronic diseases and poor physical performance in older age: cross-sectional analysis from the International Mobility. BMC public Health. 14(1):293. 24684705 10.1186/1471-2458-14-293 PMC3977880

[12] Roberts HC, Denison HJ, Martin HJ, Patel HP, Syddall H, et al (2011) A review of the measurement of grip strength in clinical and epidemiological studies: Towards a standardised approach. Age Ageing 40(4):423–429. 10.1093/ageing/afr051 21624928

[13] Fess E (1992) Grip Strength, 2nd edition Chicago: American Society of Hand Therapists.

[14] Guralnik JM, Ferrucci L, Simonsick EM, Salive ME, Wallace RB (1995) Lower-extremity function in persons over the age of 70 years as a predictor of subsequent disability. N. Engl. J. Med. 332(9):556–561. 7838189 10.1056/NEJM199503023320902 PMC9828188

[15] Guralnik JM, Simonsick EM, Ferrucci L, Glynn RJ, Berkman LF, et al (1994) A Short Physical Performance Battery assessing lower extremity function: Association with self-reported disability and prediction of mortality and nursing home admission. J. Gerontol. 49:M85–M94. 8126356 10.1093/geronj/49.2.m85

[16] Harlow SD, Gass M, Hall JE, Lobo R, Maki P, et al (2012) Executive summary of the Stages of Reproductive Aging Workshop + 10: addressing the unfinished agenda of staging reproductive aging. *J*. Clin. Endocrinol. Metab. 97(4):1159–68. 22344196 10.1210/jc.2011-3362 PMC3319184

[17] Kuh D, Bassey EJ, Butterworth S, Hardy R, Wadsworth MEJ (2005) Grip strength, postural control, and functional leg power in a representative cohort of British men and women: associations with physical activity, health status, and socioeconomic conditions. J. Gerontol. A. Biol. Sci. Med. Sci. 60(2):224–231. 15814867 10.1093/gerona/60.2.224

[18] Brazil, Salário mínimo nominal e necessário (2014) Available: http://www.dieese.org.br/analisecestabasica/salarioMinimo.html. Accessed 2014 Jul 3.

[19] Sallinen J, Stenholm S, Rantanen T, Heliövaara M, Sainio P, et al (2010) Hand-grip strength cut points to screen older persons at risk for mobility limitation. J. Am. Geriatr. Soc. 58(9):1721–6. 10.1111/j.1532-5415.2010.03035.x 20863331 PMC2946262

[20] Hergenroeder AL, Brach JS, Otto AD (2011) The Influence of Body Mass Index on Self-report and Performance-based Measures of Physical Function in Adult Women. Cardiopulm Phys Ther J 22(3):11–20. 21886476 PMC3163413

[21] WHO. Global database on Body Mass Index. Available: http://apps.who.int/bmi/index.jsp?introPage=intro_3.html. Accessed 2014 Jul 7.

[22] Whitworth JA (2003) World Health Organization (WHO)/International Society of Hypertension (ISH) statement on management of hypertension. J. Hypertens. 21(11):1983–1992. 14597836 10.1097/00004872-200311000-00002

[23] Kurina LM, Gulati M, Everson-Rose SA, Chung PJ, Karavolos K, et al (2004) The effect of menopause on grip and pinch strength: results from the Chicago, Illinois, site of the Study of Women’s Health Across the Nation. Am. J. Epidemiol. 160(5):484–491. 15321846 10.1093/aje/kwh244

[24] Iannuzzi-Sucich M, Prestwood KM, Kenny AM (2002) Prevalence of sarcopenia and predictors of skeletal muscle mass in healthy, older men and women. J. Gerontol. A. Biol. Sci. Med. Sci. 57(12):M772–777. 12456735 10.1093/gerona/57.12.m772

[25] Van Geel TA, Geusens PP, Winkens B, Sels JP, Dinant GJ (2009) Measures of bioavailable serum testosterone and estradiol and their relationships with muscle mass, muscle strength and bone mineral density in postmenopausal women: a cross-sectional study. Eur. J. Endocrinol. 160(4):681–687. 10.1530/EJE-08-0702 19174532

[26] Phillips SK, Rook KM, Siddle NC, Bruce SA, Woledge RC (1993) Muscle weakness in women occurs at an earlier age than in men, but strength is preserved by hormone replacement therapy. Clin. Sci. (Lond). 84(1):95–98. 8382141 10.1042/cs0840095

[27] Skelton DA, Phillips SK, Bruce SA, Naylor CH, Woledge RC (1999) Hormone replacement therapy increases isometric muscle strength of adductor pollicis in post-menopausal women. Clin. Sci. (Lond). 96(4):357–364. 10087242

[28] Messier V, Rabasa-Lhoret R, Barbat-Artigas S, Elisha B, Karelis AD, et al (2011) Menopause and sarcopenia: A potential role for sex hormones. Maturitas. 68(4):331–336. 10.1016/j.maturitas.2011.01.014 21353405

[29] Strand BH, Cooper R, Hardy R, Kuh D, Guralnik J (2011) Lifelong socioeconomic position and physical performance in midlife: results from the British 1946 birth cohort. Eur. J. Epidemiol. 26(6):475–483. 10.1007/s10654-011-9562-9 21416275 PMC3246593

[30] Hansen ÅM, Andersen LL, Skotte J, Christensen U, Mortensen OS, et al (2014) Social class differences in physical functions in middle-aged men and women. J. Aging Health. 26(1):88–105. 10.1177/0898264313508188 24584262

[31] Sousa ACPA, Guerra RO, Thanh Tu M, Phillips SP, Guralnik JM, et al (2014) Lifecourse Adversity and Physical Performance across Countries among Men and Women Aged 65–74. PLOS ONE. 9(8):e102299 10.1371/journal.pone.0102299 25101981 PMC4125146

[32] Murray ET, Hardy R, Strand BH, Cooper R, Guralnik JM, et al (2011) Gender and life course occupational social class differences in trajectories of functional limitations in midlife: findings from the 1946 British birth cohort. J. Gerontol. A. Biol. Sci. Med. Sci. 66(12):1350–1359. 10.1093/gerona/glr139 21860018 PMC3210957

[33] Gomes CDS, Maciel ACC, Freire ADNF, Moreira MA, Ribeiro MDO, et al (2014) Depressive symptoms and functional decline in an elderly sample of urban center in Northeastern Brazil. Arch. Gerontol. Geriatr. 58(2):214–218. 10.1016/j.archger.2013.10.009 24256975

[34] Tseng LA, El Khoudary SR, Young EA, Farhat GN, Sowers MF, et al (2012) The association of menopause status with physical function: the Study of Women’s Health Across the Nation. Menopause. 19(11):1186–1192. 10.1097/gme.0b013e3182565740 22760087 PMC3526111

